# Antecedents and Consequences of Smoking Cessation Intention in the Context of the Global COVID-19 Infodemic

**DOI:** 10.3389/fpubh.2021.684683

**Published:** 2021-08-23

**Authors:** Guangchao Charles Feng, Shan Zhu, Xinshu Zhao

**Affiliations:** ^1^College of Communication, Shenzhen University, Shenzhen, China; ^2^Department of Communication, University of Macau, Macau, China

**Keywords:** antismoking, TPB, HBM, COVID-19, infodemic, China

## Abstract

A growing body of scientific studies has been published to inform responses to the ongoing coronavirus pandemic, and some have claimed that cigarette smoking has a beneficial or mixed effect on the prevention and treatment of COVID-19. The presentation of such findings, unfortunately, has created an infodemic. This study integrated the theory of planned behavior and the health belief model and incorporated findings on addiction from the medical literature to predict cessation intention and support for tobacco control measures in the context of the COVID-19 infodemic. The study found that cessation intention partially mediated the effect of perceived severity and fully mediated the effects of perceived benefits, self-efficacy, and addiction on support for control measures. In addition, a positively-valenced message of the effect of smoking on the prevention and treatment of COVID-19 vs. a mixedly-valenced message was significant in predicting cessation intention, and the positively-valenced message of smoking indirectly predicted support for tobacco control measures. Perceived susceptibility, barriers, and subjective norms, however, exerted neither direct nor indirect effects on the two outcome variables.

## Introduction

Scientists worldwide have been working to find risk factors and therapeutics for COVID-19 since SARS-CoV-2 was identified. Therefore, while the effect of cigarette smoking on COVID-19 has been widely studied, unfortunately, the conclusions have been mixed and even contradictory. This trend is unfortunate because the dissemination of confusing findings through media reporting, although not intended to be harmful by the media, has not only undermined years of public health efforts to curb tobacco use but also resulted in chaos during the pandemic (1). Confusing information or misinformation circulated in society during an epidemic is defined as an infodemic by WHO (2). The infodemic is believed to have contributed to persistence of the coronavirus pandemic, as people under the influence of the infodemic tend to downplay the risk, not trust public health experts, and eventually fail to comply with the recommended health practices (2–5).

## Literature Review and Hypotheses

### Determinants of Health Behavioral Intentions

Numerous studies have examined why people do not give up cigarette smoking even though tobacco use is a major risk factor for more than 20 different types or subtypes of cancer (6). The determinants of such health behaviors or behavioral intentions have been extensively studied based on a variety of theories, most notably the theory of reasoned action (TRA) (7, 8); the theory of planned behavior (TPB) (9), which adds perceived behavioral control (PBC) to the TRA; social cognitive theory (SCT) (10); and the integrative model (IM) (11, 12) integrating the TRA, the TPB, and SCT [also see (13)]. The TRA and TPB have been widely employed in antismoking research [e.g., (14–17)] and generally have received empirical support.

Although all of the abovementioned theories have gained currency in their own right, they were essentially derived from expectancy-value theory (EVT) (18–24). EVT postulates that certain behavior is determined by two factors, i.e., expectancies (the likelihood of an outcome to be achieved through the behavior) and values (the desirability of the outcome).

Several theories or models that build upon the expectancy-value theory elaborate general expectancies in the TRA and the TPB into specific beliefs. They include the health belief model (HBM) (25) and its extension (26) and two models that are very similar to the extended HBM but have different formulations of the processes, i.e., protection motivation theory (PMT) [(27), p. 104)] [for a review on the differences between the PMT the HBM, see (28)] and the extended parallel process model (EPPM) (29, 30). In view of the similarities among these three models and their commonalities with the TPB, we formulate relevant hypotheses based on the original HBM.

### The Health Belief Model

The HBM hypothesizes that health behaviors are influenced by four kinds of health beliefs, i.e., perceived susceptibility (PSUS), perceived severity (PSES), perceived benefits (PBEN), and perceived barriers (PBAR), as well as cues to actions (CTA) (25). The HBM has been widely used to study smoking behavior (31, 32).

#### Health Beliefs

PSUS, PSES, PBEN, and PBAR refer to four specific beliefs that fall into the category of outcome expectancies. Numerous studies have consistently found that a higher level of risk appraisal of the outcome (PSUS and PSES), a higher level of PBEN and a lower level of PBAR to avoiding the outcome are associated with a higher level of likelihood of compliance with recommended health practices (31, 32). PSUS [e.g., (33, 34)], PSES [e.g., (35, 36)], PBEN [e.g., (37)], and PBAR [e.g., (31, 38)] have been examined and supported in many antismoking studies. Consequently, four interrelated hypotheses are proposed below:

H1a: People who perceive stronger susceptibility to becoming ill due to smoking are more likely to quit smoking.

H1b: People who perceive a stronger severity of the consequences of smoking are more likely to quit smoking.

H1c: People who perceive stronger benefits of smoking are less likely to quit smoking.

H1d: People who perceive stronger barriers to not smoking are less likely to quit smoking.

#### Cues to Action

CTA can be either internal (e.g., experience of malaise or symptoms) or external (e.g., public health media campaigns or doctor recommendation) to people of concern, and they trigger readiness to adopt a certain health behavior (39, 40). The positive effect of internal CTA on health intentions has been reported in prior studies (41–43). Consequently, the following hypotheses are proposed:

H2: The higher the internal CTA are, the more likely people are to quit smoking.

The external cues that people encounter are more varied than internal cues [for a review, see (44)]. Some studies [e.g., (45)] confirmed the hypothesized effect of external CTA on smoking cessation intention, but others [e.g., (46)] did not. In the case of the relationship between smoking and COVID-19, contradictory findings have been first reported in many medical journals and further publicized by mass media (47). Some studies (48–50), including a meta-analysis (51), concluded that smokers were more likely than nonsmokers to contract SARS-CoV-2 and to have more severe symptoms. Nevertheless, other studies (52, 53), did not confirm that smoking was a risk factor for COVID-19. In addition, a few studies (54–57) even claimed that smoking was a protective factor against COVID-19.

Notwithstanding the paramount importance, the actual impact of mixed findings with respect to the relationship between smoking and COVID-19 on cessation intention has not yet been empirically examined. A research question is hence raised below:

RQ1: Are there differences in the effects of three types of message valence (positive, negative, and mixed findings on the relationship between smoking and COVID-19) and the control condition on cessation intention and tobacco control measures?

### Back to the Original—Takeaways From Expectancy-Value Theory

As mentioned above, the HBM was derived from EVT but differs from EVT primarily with regard to specific behavioral beliefs replacing the general beliefs. In addition, compared to the more general EVT model (e.g., the TPB), the original HBM ignores subjective norms and self-efficacy (SEF).

#### Self-Efficacy

Originally proposed in social learning theory, self-efficacy is a “belief in one's capabilities and effectiveness to organize and execute the courses of action required in performing specific tasks” (10, 58–60). SEF is included in the TRA and the TPB but is referred to as PBC. PBC is an individual's perceived extent of control over performance and is jointly determined by control beliefs related to the presence of factors that may affect the performance of a behavior and the perceived power of situational and internal factors to inhibit or facilitate the performance of the behavior (7–9). In the IM, Fishbein (11, 12) referred to perceived power as SEF. SEF was later included in the extended HBM (26) and explicitly formulated in both PMT and the EPPM.

SEF has been hypothesized to positively affect behavioral intentions and has received empirical support in numerous antismoking studies (61–64). Consequently, we propose the following hypothesis:

H3: The higher SEF is, the higher smoking cessation intention is.

#### Subjective Norms

The TRA and the TPB underscore the importance of perceived social norms in affecting intention. Sullivan et al. (65) included subjective norms in the HBM and found that it affected the intention to participate in premarital prevention programs. In antismoking studies, many studies [e.g., (66, 67)] have concluded that tobacco denormalization (communicating that smoking is not a normal activity in our society) is a successful population-level strategy for fighting smoking [also see (68)] [cf. (69)]. Many studies have also found individual-level subjective norms to significantly influence smoking cessation intention (62, 64, 70–72). Consequently, we hypothesize as follows:

H4: The higher subjective norms are, the higher smoking cessation intention is.

### Smoking Addiction

According to the IM, the effect of smoking or nicotine addiction on cessation intention may be at best close to that of habit if addiction is considered a kind of past habitual behavior (73). However, addiction is more than a kind of habit, with the literature (74–77) suggesting that addiction, as a chronic disorder, might require long-term neurobiological and behavioral treatment, as well as counseling. As mentioned above, smokers smoke for different reasons [for a review of the theories of addiction, see Newton et al. (78)] and have varying degrees of addiction (79), ranging from light (rare social smokers) to severe (dependent due to nicotine withdrawal syndrome). Regardless of the reasons for addiction, the degree of addiction has been found to be a strong predictor of quitting smoking in many prior studies (80–82). Hence, we propose the following hypothesis:

H5: The higher smoking addiction is, the less likely people are to quit smoking.

### Consequences of Smoking Cessation Intention—Support for Stricter Regulations

Although numerous factors influencing support for government control of smoking have been examined (83–87), there is a lack of a coherent and sound theoretical framework. Ling et al. (88) argued that support for anti-tobacco industry action together with mistrust of tobacco companies constitute the two major factors of denormalization attitudes. We reason that support for control measures requires a heightened or more in-depth awareness of health behaviors beyond one's original attitude toward health behaviors. Previous studies (88, 89) found that support for tobacco control was positively related to intention to quit. The present study hence adheres to the EVT framework and hypothesizes that such a supportive attitude is, on the one hand, predicted by health behavioral intention and, on the other hand, both directly and indirectly affected by specific health beliefs, cues to action, subjective norms, self-efficacy and addiction [also see (90)]. That is, the variable of cessation intention also acts as a mediator between the predictors of cessation intention and support for control measures. Consequently, a series of related hypotheses are proposed below:

H6a: There is a positive relationship between cessation intention and support for tobacco control measures.

H6b: There is a positive relationship between PSUS to becoming ill due to smoking and support for tobacco control measures.

H6c: There is a positive relationship between the PSES of smoking consequences and support for tobacco control measures.

H6d: There is a negative relationship between the PBEN of smoking and support for tobacco control measures.

H6e: There is a positive relationship between PBAR to not smoking and support for tobacco control measures.

H6f: The higher subjective norms are, the more likely people are to support tobacco control measures.

H6g: The higher self-efficacy is, the more likely people are to support tobacco control measures.

H6h: The higher smoking addiction is, the less likely people are to support tobacco control measures.

H7: The associations between the predictors (shown in H6b through H6h) and support for tobacco control measures are partially mediated by cessation intention (because the predictors in H6b through H6h also directly predict tobacco control).

There is one additional research question regarding the effects of external cues to action.

RQ2: Are there differences in the direct and indirect effects of the three types of message valences (positive, negative, and mixed findings on the relationship between smoking and COVID-19) and the control condition on support for tobacco control measures?

## Methods

### Participants

The participants were recruited through a paid research panel hosted by the online survey platform “Questionnaire Star” in China in September 2020. All the participants were required to be smokers according to the clinical guidelines of the CDC (91). We excluded 2,412 participants who failed to meet the quality control conditions (were nonsmokers, did not correctly answer the question regarding the valence of the stimulus, or answered the reverse-coded scale questions in the same way as the other questions). The valid sample comprised 700 participants, and the incidence rate was 22%.

### Procedures

At the beginning of the questionnaire, a consent form was presented to the participants. If the participants agreed to the terms and conditions, they could subsequently begin to answer questions. However, the questionnaire was terminated immediately if they objected to the consent form. The online questionnaire proceeded page by page, and the participants could not return to a page once they had progressed beyond it. The questionnaire consisted of the following sections: a pretest (demographics were inquired), the treatment (stimulus), a manipulation check, and a posttest (health beliefs, evaluations, and concerns, intention to quit smoking and support for tobacco control measures). After the participants submitted the questionnaire, they were debriefed regarding the veracity of stimuli and were advised to consult with the WHO guidelines, which were accessible through a link provided to information about the relationship between smoking and COVID-19.

### Stimuli

The study manipulated external CTA, specifically, the valence of the relationship between smoking and COVID-19. There were three treatment groups based on the message valences received, i.e., that smoking is beneficial to prevention and the treatment of COVID-19 (the positively-valenced message group), that smoking increases one's risk of contracting COVID-19 and worsens the severity of COVID-19 (the negatively-valenced message group), and that smoking may have both negative and positive effects on the risk of infection and treatment of COVID-19 (the mixedly-valenced message group). A total of 600 subjects were randomly assigned to the three groups. An additional 100 subjects were also randomly assigned to a control group in which no stimulus was presented. The four groups were dummy coded for three predictors, with the mixedly-valenced message group as the reference category, because we aim to examine the effect of COVID-19 Infodemic on smoking cessation. The stimuli are stored at the online appendix (https://ndownloader.figshare.com/files/28524306?private_link=04d6bc1646c1eb6f7278).

A question was asked to determine if the manipulation was successful: “According to the reading material, is smoking bad or good for the prevention and treatment of COVID-19?” Participants who answers were inconsistent with the group to which they belonged were disqualified from the study.

### Measures

All factors but those stated otherwise below were measured using a 7-point scale [for an explanation of using a 7-point scale, see (92)] anchored with “strongly disagree” and “strongly agree” (there was also a “not applicable” category). All the measurement scales were subjected to principal component analysis (PCA) with varimax rotation and reliability tests using Cronbach's α.

Before PCA, the Kaiser–Meyer–Olkin (KMO) test of sampling adequacy and Bartlett's test of sphericity were conducted for all scales. The results were above the recommended cut-off values [the KMO value was above the acceptable level of 0.6 (93) (p. 84), and the sphericity test supported the rejection of the null hypothesis]. The number of extracted factors in PCA was finalized based on the eigenvalue-greater-than-one rule (94). Subsequent to PCA, the factor scores of the measurement scales were estimated and used in the path analysis to test the hypotheses.

#### Endogenous Variables

##### Support for Tobacco Control Measures

This construct was measured with five items that we developed by consulting with authoritative sources (95–97). The items were as follows: “Cigarette packages should contain graphic warnings of illness and death caused by smoking,” “Smoking in public places should be punished with the same measures as those implemented in foreign countries, i.e., a fixed penalty of at least 1,000 RMB (~153 US dollars),” “The price of tobacco products should increase substantially,” “All forms of commercial promotion activities in relation to tobacco products should be completely banned,” and “The punitive measures for smoking in public places during the COVID-19 pandemic should be strengthened.” PCA yielded one factor that explained 50% of the variance in the items. The factor loadings were 0.66, 0.80, 0.67, 0.66, and 0.74, and the Cronbach's α was 0.75.

##### Intention to Quit Smoking

This construct was measured with the following three items, all of which were prefaced with “Under the threat of COVID-19…”: “…you will stop smoking immediately,” “…you will gradually stop smoking in the next week,” and “…you will quit smoking in the next 30 days.” PCA yielded one factor that explained 67% of the variance in the items. The factor loadings were 0.82, 0.91, 0.87, and −0.65, and the Cronbach's α was 0.83.

#### Exogenous Variables

##### Control Variables

The control variables were mainly sociodemographic variables, including gender (30.39% of the participants were women), age (44.22, 47.79, 6.7, and 1.28% were 18–30, 31–40, 41–50, and 51–60 years old, respectively), education level (84.74% had a college degree), and monthly income (14.12, 46.79, and 25.96% earned below 5,000 RMB, 5,001–10,000 RMB, and 10,001–15,000 RMB, respectively, and the rest earned more than 15,000 RMB).

##### Smoking Addiction and Nicotine Dependence

This construct was measured using six items based on previously developed scales (98–100): “The first cigarette in the morning is the most difficult for you to quit,” “It is difficult for you not to smoke in public places where smoking is prohibited,” “You still smoke even if you are very sick,” “Once you stop smoking for a few hours, you feel restless and yawn,” “You smoke when you feel unhappy/depressed/sad,” and “You will accept an invitation to smoke together.” PCA yielded one factor that explained 56% of the variance in the items. The factor loadings were 0.74, 0.71, 0.72, and 0.81, and the Cronbach's α was 0.73.

##### Subjective Norms

Subjective norms were measured with five items asking if the following significant others of the respondent smoked: “parents,” “brothers and sisters,” “spouse/partner,” “your closest friend(s),” and “general friends/colleagues/classmates.” The response options for these items included “never,” “very rarely,” “seldom,” “occasionally,” “often,” “frequently,” and “very frequently” (there was also an “NA” option).

PCA yielded two factors (family and peer norms) that explained 58% of the variance in the items. The factor loadings were 0.84 and 0.88 on the family norms factor (the loadings of the remaining items were below 0.11) and 0.75, 0.71, and 0.73 on the peer norms factor (the loadings of the remaining items were below 0.10), and the Cronbach's α coefficients were 0.70 and 0.68 for family norms and peer norms, respectively.

##### Perceived Susceptibility to Becoming Ill

This construct was measured with four items based on a previous scale (101). The participants were asked, “Do you suspect that you may have the following health problems due to smoking?” They were then presented with the following items: “Pneumonia/lung cancer and other lung health problems,” “Heart health problems such as angina pectoris/coronary heart disease,” “Respiratory health problems such as cough/asthma/bronchitis,” and “Stroke.” PCA yielded one factor that explained 63% of the variance in the items. The factor loadings were 0.80, 83, 0.78, and 0.75, and the Cronbach's α was 0.80.

##### Perceived Severity of Smoking

This construct was measured with four items based on a previous scale (102): “Smoking can increase the risk of type 2 diabetes by 40%,” “Smoking can double the risk of stroke,” “Smoking, as well as passive second-hand smoking, can cause serious cardiovascular diseases, such as hypertensive heart disease, rheumatic heart disease, aneurysm, endocarditis, etc.,” and “Smoking, as well as passive second-hand smoking, can cause a variety of serious respiratory diseases, such as tracheitis, bronchitis, obstructive lung disease, and even lung cancer.” PCA yielded one factor that explained 55% of the variance in the items. The factor loadings were 0.75, 0.79, 0.80, and 0.62, and the Cronbach's α was 0.76.

##### Perceived Benefits of Smoking

This variable was measured with four items developed with reference to Li and Kay (103): “Smoking makes you more attractive,” “Smoking helps you relieve stress,” “Smoking makes you feel happy and relaxed,” and “Smoking makes your mind agile.” PCA yielded one factor that explained 58% of the variance in the items. The factor loadings were 0.78, 0.79, and 0.72, and the Cronbach's α was 0.67.

##### Perceived Barriers to Quitting Smoking

This construct was measured with three items developed with reference to Li and Kay (103): “Restraining yourself from smoking makes you unable to concentrate,” “Restraining yourself from smoking leads to alienation from smoking friends around you,” and “Restraining yourself from smoking makes you difficult in socializing with smokers.” PCA yielded one factor that explained 63% of the variance in the items. The factor loadings were 0.64, 0.86, and 0.86, and the Cronbach's α was 0.74.

##### Internal Cues to Action

This construct was measured with five items following the question “Have you experienced the following symptoms recently?”: “bad breath or yellow teeth,” “emphysema, pneumonia (including COVID-19), lung cancer and other lung-related diseases,” “cough, asthma, bronchitis, and other respiratory diseases,” “heart-related diseases such as rapid heart rate, angina pectoris, coronary heart disease, etc.,” and “stroke symptoms such as slurred speech and mental disorders.” PCA yielded one factor that explained 72% of the variance in the items. The factor loadings were 0.87, 0.80, 0.87, and 0.85, and the Cronbach's α was 0.88.

##### Self-Efficacy

A 3-item measurement scale was developed to measure self-efficacy for quitting smoking. The items included “You can stop smoking for 24 h,” “You can stop smoking for a whole week,” and “You can stop smoking for more than 1 month.” PCA yielded one factor that explained 82% of the variance in the items. The factor loadings were 0.86, 0.95, and 0.90, and the Cronbach's α was 0.88.

## Results

**Figure 1:**
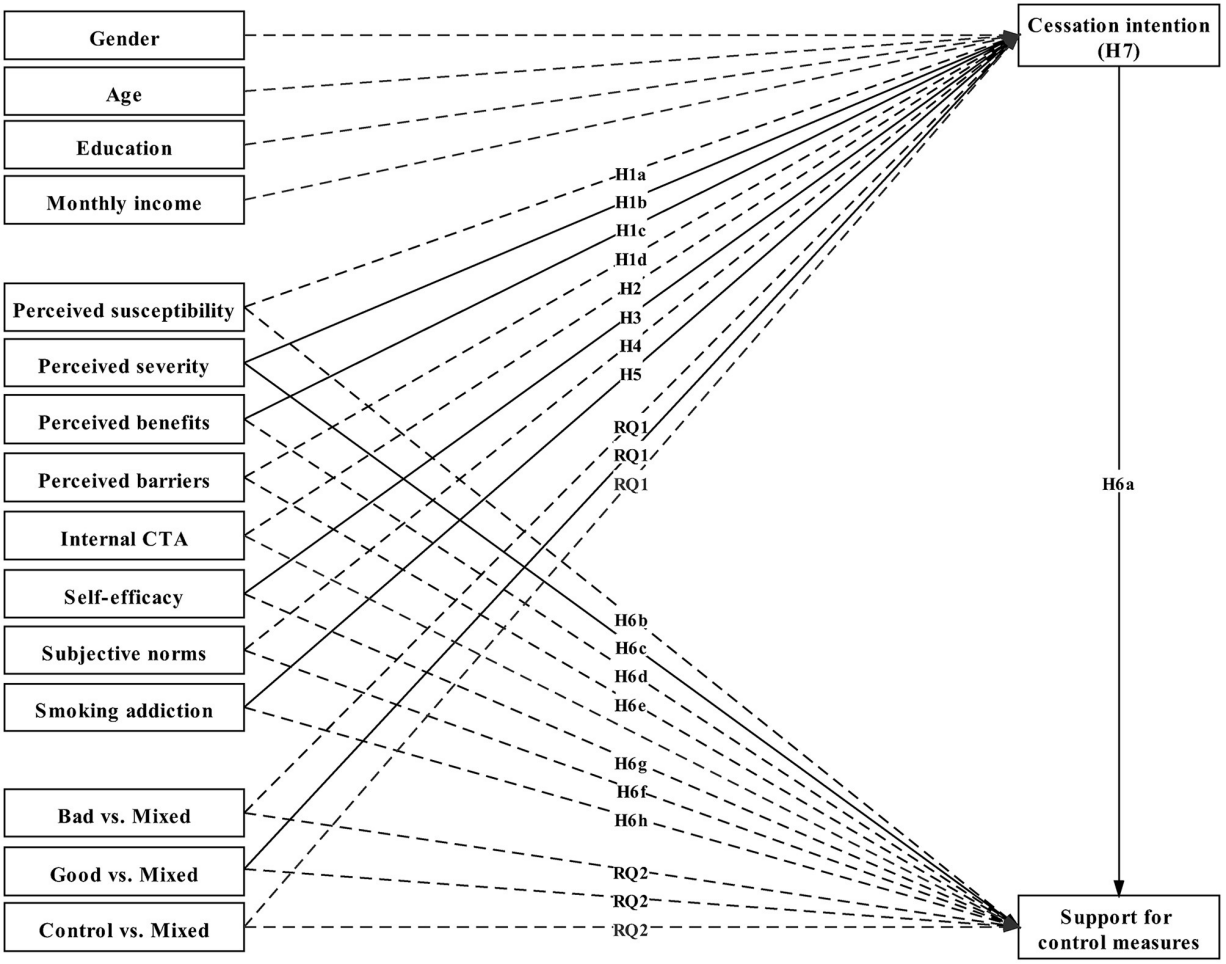
Estimation results of the proposed model (Model 1).

Table 1 shows the zero-order correlations among the variables. A series of path analyses were performed to test the hypotheses using M*plus* 8.4 (104), in which the mediation test was performed using bootstrapping with 5,000 resamples [for a discussion of problems associated with Baron and Kenny (105), see Preacher and Hayes (106)]. The hypothesized model (M1) (Figure 1) was compared with two alternative models (M2 without the direct effects of the predictors on support for control measures and M3 with self-efficacy (SEF) as the moderator for the relationships between PSUS and cessation intention and between PSES and cessation intention, as stipulated in PMT and the EPPM). The chi-square difference test between M1 and M2 was significant ($\chi_{\Delta }^{2}\;=\;55.988,\;df_{\Delta }\;=12,\;p\;<\;0.001$), in favor of M1. The model fit indices of the hypothesized model were satisfactory (χ^2^ = 8.924, *df* = 4, *p* = 0.063, CFI = 0.985, TLI = 0.879, SRMR = 0.015, and RMSEA =0.042.) Nonetheless, the chi-square difference test between M1 and M3 was not significant ($\chi_{\Delta }^{2}\;=\;-0.261,\;df_{\Delta }\;=0,\;p\;=\;1$), also in favor of M1 according to the parsimony principle. The *R*^2^ values for cessation intention and support for control measures were 0.238 and 0.214, respectively, indicating that 23.8 and 21.4% of the variance in the outcome variables, respectively, was accounted for by the predictors.

**Table 1:** Zero-order correlations.

	**Support ctl**	**Cessation int**	**Gender**	**Age**	**Edu**	**Income**	**norms_peer**	**norms_family**	**Susceptibility**	**Severity**	**Benefits**	**Barriers**	**In CTA**	**efficacy**	**Addiction**	**Bad**	**Good**
**Support ctl**																	
Cessation int	0.386																
Gender	−0.044	0.058															
Age	0.024	−0.08	−0.026														
Edu	−0.03	0.03	0.046	−0.079													
Income	0.058	−0.017	0.024	0.199	0.259												
norms_peer	0.05	−0.032	−0.278	0.057	−0.074	0.05											
norms_family	−0.041	−0.035	0.357	0.034	0.004	0.089	0.045										
Susceptibility	0.196	0.146	0.008	0.051	−0.057	−0.008	0.111	0.028									
Severity	0.302	0.226	0.046	0.062	0.033	0.031	0.019	0.054	0.474								
Benefits	−0.034	−0.17	−0.01	0.02	0.068	0.093	0.177	0.066	0.095	0.114							
Barriers	0.003	−0.067	−0.05	0.058	0.05	0.123	0.242	0.103	0.129	0.022	0.306						
In CTA	0.094	0.125	0.065	0.147	−0.047	−0.009	0.101	0.111	0.376	0.195	−0.048	0.261					
efficacy	0.174	0.394	0.097	−0.118	0.112	−0.049	−0.196	−0.027	−0.018	0.097	−0.253	−0.234	0.02				
Addiction	−0.065	−0.181	−0.07	0.076	−0.074	0.1	0.253	0.126	0.224	0.026	0.227	0.422	0.267	−0.405			
Bad	0.078	0.069	−0.088	0	0.021	−0.043	0.143	−0.014	0.019	0.015	0.049	0.023	0.083	−0.059	0.062		
Good	−0.114	−0.119	0.063	0.01	0.006	−0.078	−0.062	0.042	−0.014	0.013	−0.001	0.026	−0.015	−0.006	−0.009	−0.4	
Control	0.063	0.029	0.041	0.037	−0.034	0.056	0.005	−0.008	−0.027	0.011	−0.031	0.007	−0.028	0.012	0.002	−0.258	−0.258

Cessation intention was significantly predicted by perceived severity (β = 0.166, *p* < 0.001), perceived benefits (β = −0.112, *p* < 0.01), perceived self-efficacy (β = 0.328, *p* < 0.001), and addiction (β = −0.086, *p* < 0.05). Consequently, H1b, H1c, H3, and H5 were supported, but H1a (susceptibility), H1d (barriers), H2 (internal CTA), and H4 (subjective norms) were rejected.

Support for tobacco control measures was significantly predicted by cessation intention (β = 0.302, *p* < 0.001). The direct, indirect, and total effects of perceived severity were significant (β = 0.206, *p* < 0.001; β = 0.05, *p* < 0.001; β = 0.256, *p* < 0.001). In addition, the indirect effects of perceived benefits of smoking (β = −0.034, *p* < 0.01), self-efficacy (β = 0.099, *p* < 0.001), and addiction (β = −0.028, *p* < 0.05) were also significant. That is, cessation intention partially mediated the effect of perceived severity, whereas the full mediation for it fully mediated the effects of perceived benefits, self-efficacy, and addiction. Nevertheless, there were neither direct nor indirect effects for perceived susceptibility, barriers, and subjective norms. Consequently, H6a (prediction of support for tobacco control measures from cessation intention) and H6c (prediction for tobacco control measures from perceived severity) were supported, but the rest of the H6 sub-hypotheses were rejected. As only three indirect effects were significant, H7 (mediations) was partially supported.

Demographic variables, perceived susceptibility, and subjective norms (both family and peer norms) did not play any role (either direct or indirect effects) in predicting any of the endogenous variables. Consequently, the hypotheses regarding susceptibility (H1a and H6b) and subjective norms (H4 and H6f) were rejected.

Regarding the RQs, the positively-valenced message vs. the mixedly-valenced message was significant in predicting cessation intention (β = −0.091, *p* < 0.05). Nevertheless, only an indirect effect of the positively-valenced message of smoking was found for the prediction of support for tobacco control measures (see Table 2 for all of the estimation results). The experimentally manipulated external CTA had the expected effects on cessation intention (*M*_*negative*_ = 0.109, *SD*_*negative*_ = 1.009; *M*_*control*_ = 0.071, *SD*_*control*_= 1.075; *M*_*positive*_= −0.188, *SD*_*positive*_ = 0.962; and *M*_*mixed*_= 0.044, *SD*_*mixed*_ = 0.971) and support for tobacco control (*M*_*negative*_ = 0.123, *SD*_*negative*_ = 0.964; *M*_*control*_ = 0.153, *SD*_*control*_= 1.017; *M*_*positive*_= −0.179, *SD*_*positive*_ = 0.967; and *M*_*mixed*_= −0.020, *SD*_*mixed*_ = 1.036), but only the comparison between the positively-valenced message group and the mixedly-valenced message group was significant. That is, people who read a message that describes smoking as beneficial for the prevention and treatment of COVID-19 are less likely to stop smoking than those who read a message that states smoking as having a mixed effect on the prevention and treatment of COVID-19. Two separate ANOVAs together with Tukey's multiple comparisons of means further revealed that people who read a message that describes smoking as good for the prevention and treatment of COVID-19 are less likely to stop smoking and to support tobacco control measures than those who read a message that states smoking has a detrimental effect [*F*_(3, 696)_ = 3.493, *p* = 0.015, $\eta^{2}=\;0.015,\;Mean_{diff}$ = −0.297, *p* < 0.05; *F*_(3, 696)_ = 4.019, *p* = 0.008, $\eta^{2}=\;0.017,\;Mean_{diff}$ = −0.303, *p* < 0.05].

**Table 2:** Results of model estimation.

**DV**	**IV**	**Estimate**	**Std. Error**	**Est./Std**.
Support for control measures	Cessation intention	0.302^***^	0.040	7.495
	Subjective norms (peer)	0.053	0.036	1.458
	Subjective norms (family)	−0.038	0.036	−1.059
	Perceived susceptibility	0.059	0.044	1.335
	Perceived severity	0.206^***^	0.043	4.761
	Perceived benefits	−0.012	0.040	−0.296
	Perceived barriers	0.031	0.043	0.717
	Internal CTA	−0.011	0.038	−0.277
	Self-efficacy	0.038	0.043	0.883
	Smoking addiction	−0.033	0.043	−0.767
	Bad vs. mixed	0.046	0.041	1.110
	Good vs. mixed	−0.044	0.042	−1.048
	Control vs. mixed	0.052	0.039	1.318
Cessation intention	Subjective norms (peer)	0.048	0.040	1.211
	Subjective norms (family)	−0.044	0.040	−1.117
	Perceived susceptibility	0.068	0.042	1.634
	Perceived severity	0.166^***^	0.041	4.100
	Perceived benefits	−0.112^**^	0.039	−2.839
	Perceived barriers	0.049	0.042	1.180
	Internal CTA	0.066	0.043	1.551
	Self-efficacy	0.328^***^	0.039	8.429
	Smoking addiction	−0.086^*^	0.043	−2.000
	Bad vs. mixed	0.053	0.041	1.303
	Good vs. mixed	−0.091^*^	0.039	−2.356
	Control vs. mixed	0.012	0.038	0.312

* *p ≤ 0.05;*

** *p ≤ 0.01;*

*** *p ≤ 0.001*.

## Discussion

Consistent with prior studies, there is a positive association between cessation intention and support for tobacco control measures. Support for control measures is the attitude toward punitive behaviors, which is different from the attitude toward (un)healthy behaviors *per se*. Therefore, support for control measures is a specific attitude that can be predicted by the intention to engage in health behaviors. The first step in gaining smokers' support for tougher regulations is to dissuade them from smoking and convince them of the threat that smoking brings.

As mentioned above, cessation intention partially mediated the effect of perceived severity and fully mediated the effects of perceived benefits, self-efficacy, and addiction on support for control measures. A perception of the severity of smoking consequences determines both cessation intention and support for punitive measures against smoking. Therefore, focusing on and publicizing the severity of smoking consequences are crucial to both induce people to quit smoking and garner their support for stricter regulations against tobacco use. Furthermore, the perceived benefits of smoking, self-efficacy, and addiction contributed to cessation intention in the hypothesized directions, and these predictors affected support for tobacco control measures through the mediator, i.e., cessation intention. Perceived benefits [and severity as mentioned above; Rogers (13); Witte (29)] increase motivation, and self-efficacy promotes ability, while addiction might undermine the opportunity to process persuasive information. Motivation, ability, and opportunity (MAO) (107) have been found to influence process levels (involvement) and subsequently attitudes and behaviors.

Perceived susceptibility (H1a), barriers (H1d), and subjective norms (H4) exerted neither direct nor indirect effects on the outcome variables. However, perceived severity not only predicted cessation intention but also directly and indirectly predicted support for control measures. Compared to susceptibility, perceived severity promotes individuals' complete knowledge of smoking hazards [see (108)] and primes them to resist the belief that they are immune to smoking-caused health risks (109) and to take seriously the risk of smoking to their health [see (76)]. This may also indirectly explain why susceptibility was not predictive of the two outcome variables. People are most likely to be desensitized by widely generalized information regarding smoking's hazardous influence on health so that they truly do not realize the severity of susceptibility [cf. (76)] [most smokers in China were aware of the smoking hazards according to (110)]. Such a phenomenon can possibly be explained by exemplification theory (111, 112), which hypothesizes that people are more easily influenced by concrete (and severe) examples rather than by general risk information [cf. (113)].

Besides, Janz and Becker (114) differentiated preventive-health behaviors (PHB) from sick-role behaviors (SRB) and found perceived susceptibility was a stronger contributor for explaining PHB than SRB, yet perceived benefits and perceived severity were strong only for SRB. Consequently, smoking cessation, as one of SRB, is only closely associated with perceived benefits and perceived severity.

The nonsignificant effect of subjective norms on cessation intention is consistent with previous studies [e.g., (34)]. This finding may demonstrate that most smokers in China are not socially driven [cf. (115)] but intrinsically driven by addiction [cf. (116)], which has consistently shown an important role in predicting cessation intention. Moreover, the nonsignificant effect of subjective norms indicates the importance of distinguishing descriptive norms, which describe what other people do and is used in the present study, from injunctive or prescriptive norms, which prescribe how people should do. Many prior studies (117, 118) have found that injunctive norms play a more important role in predicting smoking cessation than do descriptive norms. In addition, the results may also reveal that different factors prevent people from smoking than those that persuade them to quit smoking [cf. (116)]. The former (the prevention of smoking among nonsmokers), as demonstrated in the success of denormalization campaigns of antismoking, is susceptible to social influence, but the latter (cessation intention among smokers) is not. Public health media campaigns should make use of the difference in determinants between smokers and nonsmokers and develop tailored messages with effective determinants to target these two groups. This might be a practical implication of this study.

Internal CTA (H2) did not have significant associations with the outcome variables. Examining the responses to the internal CTA scale, we found that the nonsignificant effects may be attributed to the little variation among the indicators. On average, the majority of the participants (mean scores below 4, i.e., about right) did not believe that they had any health problems related to smoking, which led to optimistic bias [for a review, see (119)]. The result shows that, similar to the issue of perceived susceptibility discussed above, even experiencing mild symptoms does not necessarily cue the subject to a real imminent risk. Moreover, the experimentally manipulated external CTA in general had the expected effects on cessation intentions and support for tobacco control.

The experimental findings indicate the simple fact that scientific studies are important to the wellbeing of our society. More people have been increasing their tobacco use due to distress and other mental problems caused by the pandemic (120), and confusing findings may exacerbate an already dire situation (1). Arguably, conflicting academic findings regarding the effect of smoking on COVID-19 do much more harm than general misinformation circulated on social media because the scientists who report unconventional findings are very easy to receive publicity through mainstream news media and to become more viral on social media (3, 121). Although scientists could debate on findings, media that publicize these findings should be cautious to avoid unwittingly spreading false or pseudoscientific information. This state of affairs, nevertheless, is concerning in that many media outlets have naively circulated sensational and unconventional findings, such as those on smoking being beneficial to the prevention and treatment of COVID-19 (1, 122). Media outlets should always prioritize social responsibility based on values of a high moral ground and professionalism because the information they publish could potentially cost people's lives (1). Therefore, as a caveat, alongside any unconventional findings, media outlets must report the background of the journal and authors; the controversy surrounding the findings; the official recommendations from the WHO and/or related health authorities; and more importantly, related opposing findings. This point may constitute another practical implication of the present study.

The findings of the study have additional practical implications. The nonsignificant role of subjective norms indicates that the stigmatization of smokers (denormalization) may not be an effective strategy and may even backfire, as smokers discredit social influence in their decision to continue smoking. Antismoking efforts are a collective endeavor in a civic society, which means we need to not only publicize the knowledge using concrete examples to promote beliefs about smoking hazards (severity) and the benefits of quitting and support self-control (self-efficacy) over addiction but also generate popular support for the enforcement of stricter control measures.

This study integrated the TPB and the HBM and incorporated findings on addiction from the medical literature to predict cessation intention (see Figure 1). Furthermore, the integrated framework explaining smoking cessation was extended to predict support for tobacco control measures through the repositioning of smoking cessation as a mediator. Consequently, the study makes possible theoretical contributions by proposing an integrated theoretical framework that explains two important phenomena in health communication.

The study has limitations. First of all, the measures on the smoking cessation intention and the support for control measures are collected soon after the subjects were primed with the various stimuli. This raises the concern that the effects of stimuli may well fade away in a longer time horizon, but such a limitation regarding external validity is shared by most experimental research (123).

In addition, the respondents were recruited in China, which has the largest smoking population in the world (97, 124), is the largest cultivator of tobacco (97), and is where the coronavirus pandemic first broke out (125). Moreover, there are unique regulatory systems for the tobacco industry and social customs related to smoking in China (97). Consequently, the cultural, social, economic, and political idiosyncrasies in China require the cautious interpretation of the study findings and their generalization to other contexts. A future study reproduced in another region beyond China is needed to resolve uncertainties.

Previous studies (80, 126–128) found that smokers were more likely to be from disadvantaged social groups than nonsmokers and that those from disadvantaged social groups were less likely to quit smoking than those from more advantaged groups. This study, however, did not find predictive effects of the demographic predictors that were examined. This might be due to the limitation of the sample pool recruited from the online channel. Although, we used a paid research panel to attempt to collect a random sample covering all the provinces in China, the sample was skewed toward male well-educated youths aged 18–40 in socioeconomically developed regions.

## Data Availability Statement

The raw data supporting the conclusions of this article will be made available by the authors, without undue reservation.

## Ethics Statement

The studies involving human participants were reviewed and approved by Ethical Committee of Medical School, Shenzhen University. The patients/participants provided their written informed consent to participate in this study.

## Author Contributions

GF was responsible for research design, analysis, and write-up of the manuscript. SZ was responsible for data collection and literature review. XZ was responsible for the revision of the manuscript. All authors contributed to the article and approved the submitted version.

## Conflict of Interest

The authors declare that the research was conducted in the absence of any commercial or financial relationships that could be construed as a potential conflict of interest.

## Publisher's Note

All claims expressed in this article are solely those of the authors and do not necessarily represent those of their affiliated organizations, or those of the publisher, the editors and the reviewers. Any product that may be evaluated in this article, or claim that may be made by its manufacturer, is not guaranteed or endorsed by the publisher.
